# Selective covalent capture of a DNA sequence corresponding to a cancer-driving C>G mutation in the *KRAS* gene by a chemically reactive probe: optimizing a cross-linking reaction with non-canonical duplex structures†

**DOI:** 10.1039/c9ra08009k

**Published:** 2019-10-15

**Authors:** Xu Guo, Maryam Imani Nejad, Li-Qun Gu, Kent S. Gates

**Affiliations:** Department of Chemistry, University of Missouri 125 Chemistry Building Columbia MO 65211 USA gatesk@missouri.edu +1-573-882-6763; Department of Biochemistry, University of Missouri Columbia MO 65211 USA; Department of Bioengineering, Dalton Cardiovascular Research Center, University of Missouri Columbia MO 65211 USA

## Abstract

Covalent reactions are used in the detection of various biological analytes ranging from low molecular weight metabolites to protein–protein complexes. The detection of specific nucleic acid sequences is important in molecular biology and medicine but covalent approaches are less common in this field, in part, due to a deficit of simple and reliable reactions for the covalent capture of target sequences. Covalent anchoring can prevent the denaturation (melting) of probe–target complexes and causes signal degradation in typical hybridization-based assays. Here, we used chemically reactive nucleic acid probes that hybridize with, and covalently capture, a target sequence corresponding to a cancer-driving variant of the human *KRAS* gene. Our approach exploits a reductive amination reaction to generate a stable covalent attachment between an abasic site in the probe strand and a guanine mutation at position 35 in the *KRAS* gene sequence. Importantly, systematic variation of the probe sequence in a manner that formally introduces non-canonical structures such as bulges and mispairs into the probe–target duplex led to probes with dramatically improved cross-linking properties. An optimized abasic site-containing probe enabled simultaneous quantitative detection of both mutant and wild-type *KRAS* sequences in mixtures.

## Abstract 2

A covalent cross-linking reaction used for selective capture of a disease-relevant DNA sequence.

## Graphical abstract

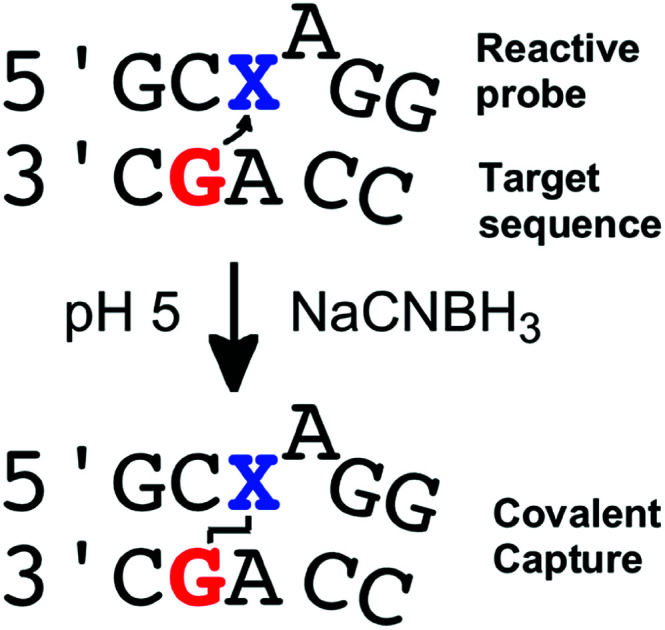

## Introduction

Many bioanalytical applications employ covalent chemistry to generate robust signals for the detection of bioactive small molecules,^1,2^ proteins,^3–5^ protein–protein complexes,^6–8^ protein–nucleic acid complexes,^9–11^ RNA–RNA interactions,^10,11^ chromatin structure,^12^ and proteins with particular functional properties.^13,14^ The detection of specific nucleic acid sequences is important in molecular biology and medicine,^15^ but covalent approaches are less common in this field, in part, due to a deficit of practical, predictable (programmable) reactions for the covalent attachment of probes to target sequences. Nucleic acid sequence detection almost universally relies on Watson–Crick pairing of a nucleic acid probe strand with the target sequence in the sample.^16^ Covalent cross-linking reactions can be used to anchor the probe strand to its target sequence thereby generating a probe–target complex that is impervious to denaturation (melting) that causes signal degradation in typical hybridization-based assays.^10,17,18^ Furthermore, sequence-specific covalent cross-linking reactions can provide increased selectivity for a particular target sequence.^19–23^ For example, we recently showed that selective cross-link formation by mechlorethamine at a C–C mismatch in a probe–target complex can be used for selective detection of a disease-relevant T→C mutation in the BRAF kinase gene sequence.^24^ In a separate study, we employed a reactive probe containing an abasic (AP) site for the selective detection of a T→A polymorphism in the *BRAF* gene.^25^ In this case, covalent cross-linking of the probe strand to the target strand involved the reaction of the AP aldehyde group in the probe with the exocyclic amino group of the adenine mutation in the target strand.^26,27^

**Scheme 1:**
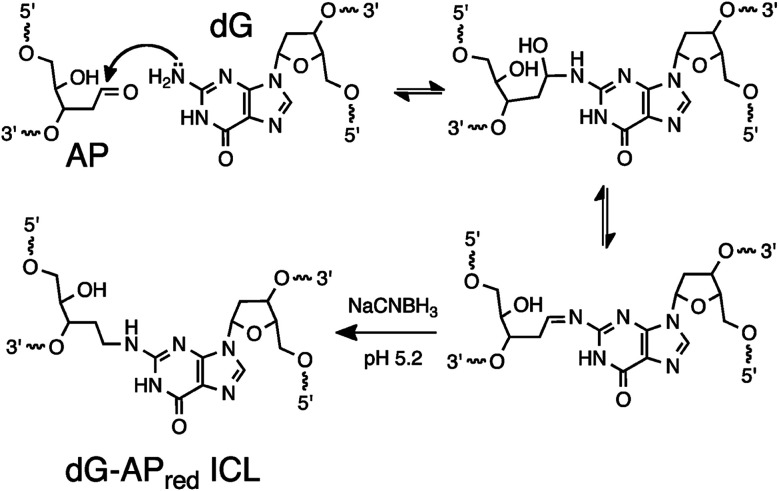
Covalent capture of target sequence *via* reaction of an AP site in the probe strand with a guanine residue in the target strand.

Even with the examples noted above, there is a need for practical, predictable reactions for sequence-selective generation of DNA–DNA cross-links. Such reactions may be useful in the detection of nucleic acid sequences^19,23–25^ and also in other diverse applications including the stabilization of nucleic acid-based materials^28^ and genome editing.^29^ Herein, we set out to explore the utility of a distinct cross-linking reaction, with the goal of expanding the options available for covalent capture of defined DNA sequences. This process exploits a reductive amination reaction between an AP aldehyde group in the probe strand and the exocyclic amino group of a guanine residue in the target sequence to generate a stable, covalently cross-linked probe–target complex (Scheme 1).^30,31^ The reaction proceeds *via* initial equilibrium formation of an imine intermediate that is subsequently reduced by sodium cyanoborohydride (NaCNBH_3_)^32^ to provide an *N*^2^-alkylguanine cross-link.^30,31^ We applied this cross-linking reaction to the detection of a DNA sequence corresponding to a C→G mutation at position 35 in the non-coding strand of the human *KRAS* gene sequence (nc35C>G, this mutation corresponds to the c35G→C transversion in the coding strand of the *KRAS* gene).^33^ This genetic variant encodes a cancer-driving G12A substitution in the *KRAS* protein.^34^ We found that the dG-AP cross-linking reaction can be employed for the selective covalent capture of the mutant *KRAS* sequence. Importantly, we showed that probe sequences deliberately designed to introduce non-canonical structures such as bulges and mispairs into the probe–target duplex can generate dramatically improved yields and selectivities for covalent capture of the target sequence.

## Results and discussion

### Selective covalent capture of a cancer-driving *KRAS* gene sequence by reaction of an AP-containing probe strand with a guanine residue in the target strand

**Fig. 1:**
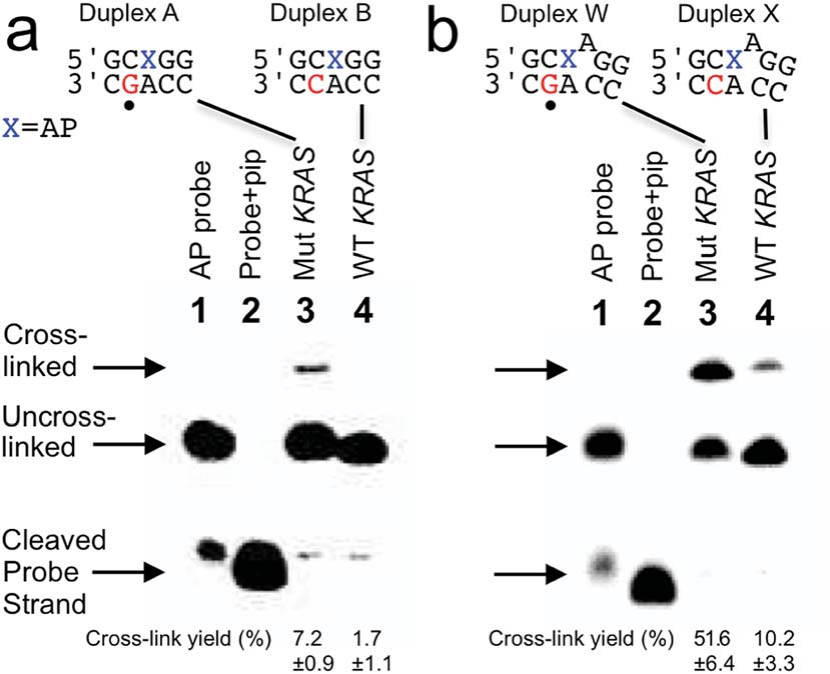
Selective covalent capture of a sequence corresponding to *KRAS* nc35C>G by probes 1 and 12. The probe strand was combined with a 21 nt “target strand” (either the WT or mutant sequence). The figure shows only the sequences flanking the reactive AP site (X) with the probe strand shown on top and the target strand on the bottom. The location of the cancer-driving mutation is marked with a dot (•) below duplexes A and W. The probe–target duplexes were incubated in sodium acetate (750 mM, pH 5.2) buffer containing NaCNBH_3_ (250 mM) for 24 h at 37 °C. Formamide loading buffer was added and the DNA in the samples resolved by electrophoresis on a denaturing 20% polyacrylamide gel. Following separation, the ^32^P-labeled oligonucleotides in the gel were visualized by phosphorimager analysis. The Panel (a): covalent capture of mutant and wild-type *KRAS* sequences by probe 1. Lane 1: 5′-^32^P-labeled AP-containing probe 1; lane 2: probe 1 treated with piperidine to induce cleavage at the AP site; lane 3: cross-link formation in the probe–mutant complex; lane 4: cross-link formation in the probe-wild type complex. Panel (b): covalent capture of mutant and wild-type *KRAS* sequences by probe 12. Lane 1: 5′-^32^P-labeled AP-containing probe 12; lane 2: probe 12 treated with piperidine to induce cleavage at the AP site; lane 3: cross-link formation in the probe–mutant complex; lane 4: cross-link formation in the probe-wild type complex. Reaction conditions and analytical methods are described in the Experimental section. Complete sequences for all probes and targets are shown in Fig. S1.† The values shown are the averages and standard deviations calculated from three or more measurements.

**Fig. 2:**
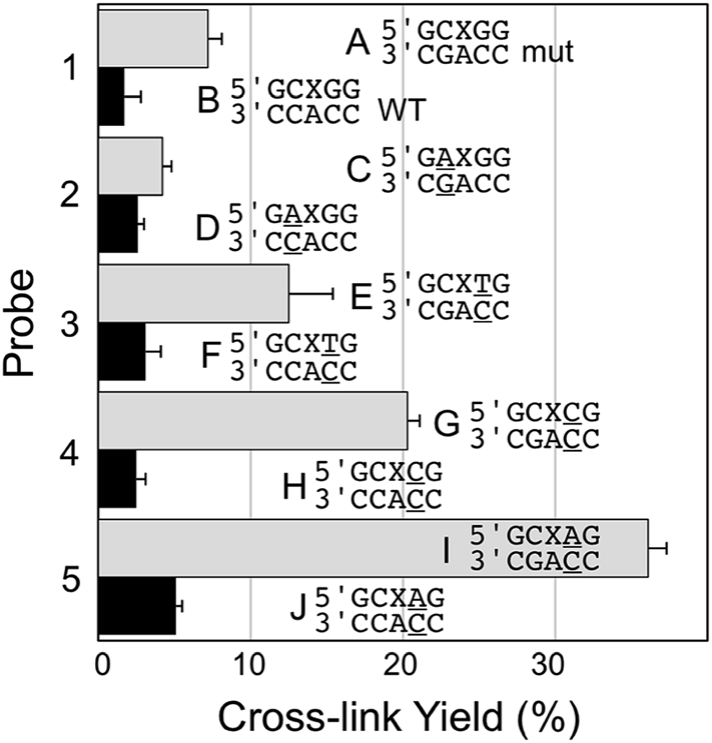
Yield and selectivity for the covalent capture (cross-linking) of mutant *versus* WT *KRAS* gene sequence by reactive, AP-containing probes (X = AP in the sequences shown) that are completely matched (probe 1) or introduce mispairs into the probe target complexes (probes 2–5, mismatches are underlined in the sequences shown above). The figure shows the sequences flanking the reactive AP site (X) with the probe strand shown on top and the target strand (either mut or WT sequence) on the bottom. The complete sequences for these duplexes are shown in Fig. S1.† The cross-link yield generated in the probe–mutant complex is depicted in the top bar of each pair (gray) and the probe–WT complex is the bottom bar in each pair (black). Cross-linking reactions and gel electrophoretic analysis were carried out as described in the legend of Fig. 1 and the Experimental section.

Our goal was to design probes in which the AP site selectively cross-linked with the guanine mutation in the nc35C>G *KRAS* gene sequence. To meet this goal we initially designed a probe sequence 1 (Fig. 1a and 2) that positioned the AP site 1 nt away from the target guanine residue in the probe–target complex. This design rested on our previous observation the AP aldehyde can forge a covalent cross-link with a guanine residue on the opposing strand offset 1 nt to the 5′-side of the AP site (duplex A, Fig. 1).^30,31^ Our approach further recognized that inclusion of the water-compatible hydride reducing agent NaCNBH_3_ in the cross-linking reaction had the potential to generate substantial yields (approximately 20%) of a chemically-stable dG-AP interstrand cross-link *via* a reductive amination reaction (Scheme 1).^31^ At the outset of the current studies, the scope and generality of this interstrand DNA cross-linking reaction was unknown, having been examined previously in only two different sequence contexts.^30,31,35^

We prepared a 21 nt, 5′-^32^P-labeled probe strand 1 containing the reactive AP site by treatment of the corresponding 2′-deoxyuracil-containing oligonucleotide with the enzyme uracil DNA glycosylase (UDG).^36–38^ Installation of the AP residue in the probes was confirmed by piperidine-induced cleavage at the AP site to generate a short 10 nt ^32^P-labeled fragment (Fig. 1a, lanes 2).^38,39^ We incubated the 5′-^32^P-labeled, AP-containing probe strand with the mutant *KRAS* target sequence in sodium acetate buffer (0.75 M, pH 5.2) containing NaCNBH_3_ (250 mM) followed by electrophoretic analysis of the ^32^P-labeled products on a denaturing 20% polyacrylamide gel. For sequence detection in a genome, standard methods can be used to obtain double-stranded genomic DNA to which a probe strand can be hybridized (for example, see ref. 40). The cross-linked, 21 bp probe–target complex was detected as a characteristic^30,31,41^ slowly-migrating band in 7.2 ± 0.9% yield (Fig. 1a). Incubation of the AP-containing probe strand with the wild-type (WT) *KRAS* sequence generated a slowly-migrating cross-link band in substantially lower yield (1.7 ± 1.1%). Thus, probe 1 provided a 4.2-fold higher signal for the mutant sequence (duplex A, Fig. 1a) over that for the WT sequence (duplex B, Fig. 1a) and a 5.5% difference in cross-link yields between mutant and WT sequences. The origin of the background signal arising from cross-link generation in duplexes lacking the target guanine residue is discussed further below.

### Probe sequences that introduce mispairs into the probe–target complex can improve selectivity and yield for covalent capture of the mutant *KRAS* sequence

Encouraged by the selective detection of mutant *KRAS* sequence by the AP-containing probe 1, we set out to determine whether alterations in the sequence of the probe that formally introduce non-canonical structures such as mispairs and bulges into the probe–target complex could improve the selectivity and yield of covalent cross-link formation with the mutant *KRAS* target sequence. Along these lines, we were inspired by our previous work showing that mispairs in probe–target complexes enabled sensitive and selective detection of T→A and T→G mutations in the *BRAF* gene sequence.^24,25^ We first examined the performance of the AP-containing probe 2 that generates a G/A mispair with the target guanine residue in the probe–mutant complex (duplex C, Fig. 2). Unfortunately, we found that this alteration in the AP probe sequence dramatically decreased both the yield and selectivity in the detection of the mutant *KRAS* sequence (4.2 ± 0.6% cross-link yield with mutant and 2.6 ± 0.4% yield for WT sequence). We next examined the effects of mispairs located on the 3′-side of the AP site, distal to the target guanine residue in the mutant sequence (probes 3–5). We found that mispairs in this location markedly improved both yield and selectivity for cross-linking of the probe to the mutant *versus* WT sequence (duplexes E–J, Fig. 2). Within this series, the best performance was obtained with probe 5 that generates an A/C mispair (36.1 ± 1.2% cross-link yield in probe–mutant duplex I, 5.1 ± 0.4% yield with probe–WT duplex J, corresponding to a 7.1-fold selectivity for target sequence, and a 31% difference in cross-link yields between mutant and WT sequences).

### Optimum selectivity and yield for covalent capture of the mutant *KRAS* sequence is achieved by an AP-containing probe strand that introduces a formal bulge into the probe–target complex

**Fig. 3:**
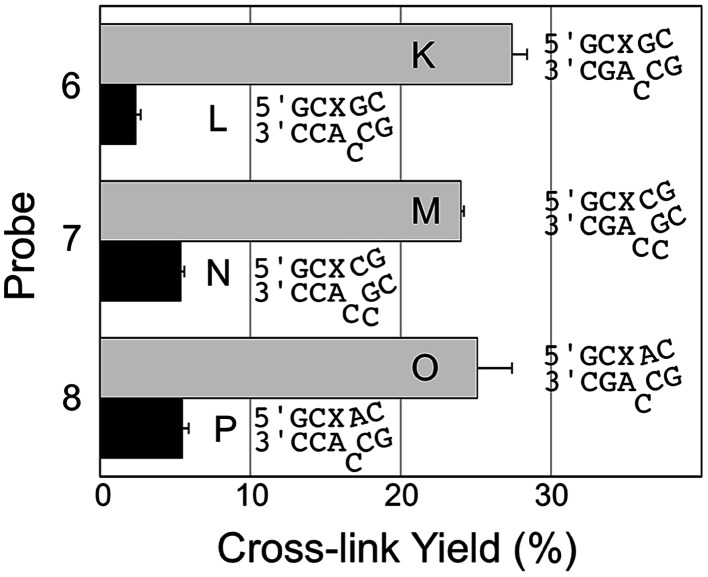
Yield and selectivity for the covalent capture (cross-linking) of mutant and WT *KRAS* gene sequences by reactive, AP-containing probes (X = AP in the sequences shown) that formally introduced bulges on the target strand of the probe–target complex. The figure shows the sequences flanking the reactive AP site (X) with the probe strand shown on top and the target strand (either mut or WT sequence) on the bottom. The complete sequences for these duplexes are shown in Fig. S1.† The cross-link yield generated in the probe–mutant complex is shown in the top bar of each pair (gray) and the probe–WT complex is the bottom bar in each pair (black). Cross-linking reactions and gel electrophoretic analysis were carried out as described in the legend of Fig. 1 and the Experimental section.

We examined the performance of AP-containing probes 6 and 7, with one or two base deletions that lead to the formal generation of 1 nt or 2 nt bulges on the target strand in the probe–target complexes (duplexes K–N, Fig. 3). These probes generated cross-link in good yield and with good selectivity for the mutant *KRAS* mutant sequence over WT, though both yields and selectivity were inferior to that provided by the best 3′-mismatch probe discussed above (Fig. 2, probe 5, duplexes I/J). Probe 8, designed to generate both a bulge and a mispair the probe–target complexes (duplexes O/P, Fig. 3), provided cross-link yields and selectivity similar to the probe that generates a formal 2 nt bulge on the target strand in the probe–target complexes (duplexes M/N, Fig. 3).

**Fig. 4:**
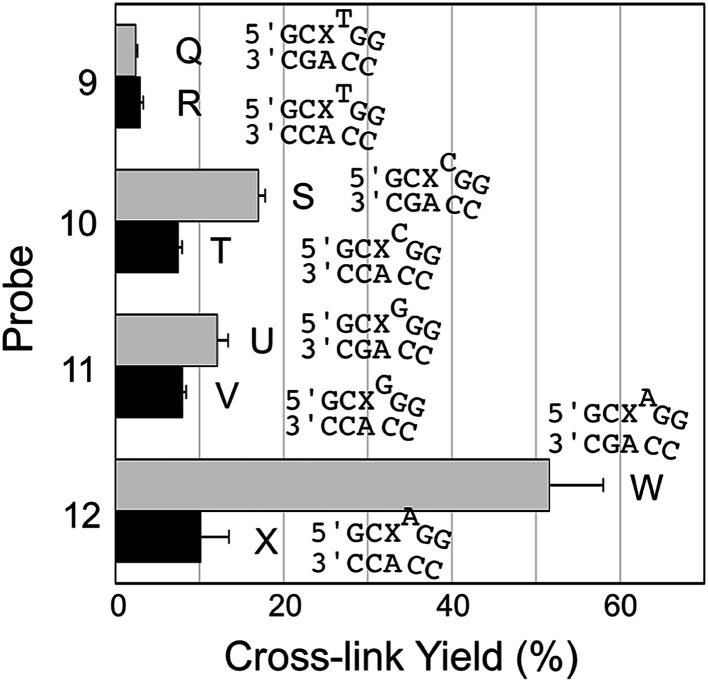
Yield and selectivity for the covalent capture (cross-linking) of mutant and WT *KRAS* gene sequences by reactive, AP-containing probes (X = AP in the sequences shown) that formally introduced bulges on the probe strand of the probe–target complex. The figure shows the sequences flanking the reactive AP site (X) with the probe strand shown on top and the target strand on the bottom. The complete sequences for these duplexes are shown in Fig. S1.† The cross-link yield generated in the probe–mutant complex is depicted in the top bar of each pair (gray) and the probe–WT complex is the bottom bar in each pair (black). Cross-linking reactions and gel electrophoretic analysis were carried out as described in the legend of Fig. 1 and the Experimental section. The performance of all twelve of the reactive probes examined in this work is compiled in Fig. S2 and Table S1.†

We examined a series of AP-containing probes 9–12 with base insertions that formally introduce a bulge on the probe strand in the probe–target complexes (Fig. 4). With the insertion of a T, C, or G into the probe, the yields of cross-link formed with the mutant *KRAS* sequence were modest and the selectivities for mutant over WT sequence rather poor (duplexes Q–V, Fig. 4). On the other hand, the insertion of an A residue into the probe (duplexes W/X) provided the best overall yield for probe–mutant cross-link formation (57.7 ± 1%), good selectivity (5.7-fold), and large yield difference (48%) over that generated with the WT sequence. A representative gel electrophoretic analysis of this cross-linking reaction is shown in Fig. 1b.

### The optimized probe 12 captures the mutant *KRAS* sequence *via* a rapid reaction with the mutant guanine residue at position 35 that does not require strict temperature control

We examined several key features of cross-link generation by the “optimized” probe 12 in duplexes W and X. Iron-EDTA footprinting experiments^31,42^ pinpointed the location of the cross-link attachment the probe–mutant duplex at the guanine mutation in the nc35C>G sequence (Fig. S3†). The reductive amination reaction rapidly generates cross-link in duplex W, giving >40% yield within 4 h and reaching a final yield of approximately 58% in about 8 h (Fig. S4†). The cross-linking reaction in duplex W is much better at pH 5.2 as opposed to pH 7, both in terms of signal intensity (yield) and selectivity for the mutant sequence over WT (Fig. S5†). This type of pH dependence is typical for a reductive amination reaction.^43^ We further demonstrated that strict temperature control is not required for the successful use of this probe. Specifically, we found that the yield and selectivity of probe 12 for mutant over WT sequence were comparable when the reaction was carried out at either 37 °C (our standard conditions above) or room temperature (24 °C, Table S1†).

We also considered the origin of the “background” cross-link that is generated in the WT duplex X lacking a target G residue at position 35. We suspected that the cross-link in duplex X may arise from a distinct process involving low yield reaction between the AP site and the directly opposing A residue.^26^ Indeed, iron-EDTA footprinting reactions on the isolated cross-link generated in duplex X provided evidence for this supposition (Fig. S6†).

### Simultaneous quantitative detection of both mutant and wild-type *KRAS* sequences in mixtures

**Fig. 5:**
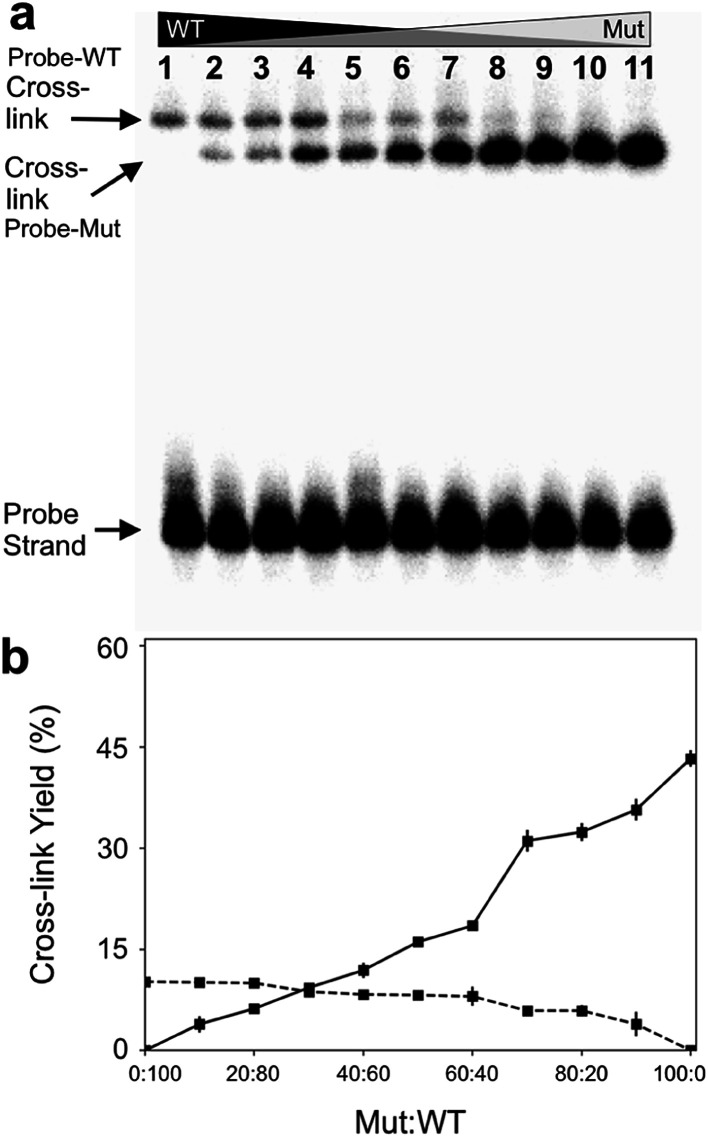
The optimized abasic site-containing probe enabled simultaneous quantitative detection of both mutant and wild-type *KRAS* sequences in mixtures with no background interference. Formation of two distinct cross-linked species can be detected in the reaction of probe 12 with mixtures containing varying ratios of mutant and WT-KRAS sequences. Cross-linking reactions and gel electrophoretic analysis were carried out as described in the legend of Fig. 1. Panel (a): gel electrophoretic analysis of cross-link formation by probe 12 with mixtures of mutant and wild-type *KRAS* sequences. The low yield cross-link formed with the wild type sequence is clearly separated from the high yield cross-link formed with the mutant *KRAS* sequence. Panel (b): a plot of the gel electrophoretic data showing the yields of probe-wild type and probe–mutant cross-links generated by incubation of probe 12 with mixtures containing varying ratios of wild-type and mutant KRAS sequence.

Finally, we examined the ability of probe 12 to selectively capture the mutant *KRAS* sequence in mixtures containing varying fractions of the mutant and WT sequences. In this experiment, we demonstrated that the cross-linked duplexes resulting from reaction of the probe with mutant and WT sequences could be cleanly separated by gel electrophoresis (Fig. 5a). This was accomplished by running the cross-linked duplexes farther into the gel than in our earlier experiments illustrated in Fig. 1. Under these analytical conditions, the relative amounts of mutant and WT *KRAS* sequences in the mixture can be separately and simultaneously measured using gel electrophoresis (Fig. 5b). Separation of the signals arising from cross-link formation with the mutant and WT sequences enables detection of the mutant *KRAS* target sequence, with no significant competing background signal (Fig. 5b). The separation of the two cross-linked duplexes is fundamentally interesting, though it must be acknowledged that gel electrophoretic analysis may not be practical for routine diagnostic use.

## Conclusions

In summary, we developed a new hybridization-induced, programmable cross-linking reaction for sequence-selective covalent capture of nucleic acids. The reactive, cross-linking probes used in these studies are prepared in a simple, one-step procedure from inexpensive commercial reagents and achieve exquisite specificity for a particular target sequence under isothermal assay conditions that do not require strict temperature control. We demonstrated the utility of these chemically reactive AP-containing probes for the covalent capture of a DNA sequence corresponding to a nc35C>G mutation in the human *KRAS* gene sequence. The covalent cross-linking reaction generates a chemically-stable, probe–target complex that is immune to thermal denaturation.^31,35,44^

The first-generation probe designed to generate a fully base-paired probe–mutant complex provided modest yields for covalent capture of the mutant *KRAS* target sequence. However, systematic variations of the probe sequence designed to introduce non-canonical mispairs and bulged structures into the probe–target duplexes significantly improved performance with respect to both signal intensity (yield) and selectivity toward the mutant *KRAS* sequence over the wild-type sequence. The three-dimensional structures of these probe–target duplexes are not known, but presumably mispairs and bulges produce dynamic complexes^45,46^ that can better accommodate distortions required for covalent cross-link formation.

Gel electrophoretic analysis of an optimized, ^32^P-labeled, abasic site-containing probe enabled simultaneous quantitative detection of both mutant and wild-type *KRAS* sequences in mixtures with no background interference (Fig. 5). While gel electrophoretic analysis may not be applicable to the clinical setting, the general approach described here for sequence-selective covalent capture could be adapted for use with other detection methods including fluorescence spectrometry,^16,47^ UV-vis spectrometry,^48^ capillary electrophoresis,^49^ nanopore technology,^24,25,50^ or electrochemistry.^51^

Development of new strategies for sequence-selective covalent capture of nucleic acids has the potential to inspire and enable significant new applications. The cross-linking chemistry developed in this work, along with our previous results involving the detection of cross-linked DNA using a protein nanopore,^50^ enables PCR-free, single-molecule detection of target DNA sequences. In other areas, programmable cross-linking reactions like the one described here may ultimately find uses in the other types of nucleic acid-based sensors and in the construction of functional nucleic acid materials.^28^

## Experimental section

### Materials and methods

DNA oligonucleotides were purchased from Integrated DNA Technologies (IDT, Coralville, IA), [γ-^32^P]-ATP was purchased from PerkinElmer Life Science, uracil DNA glycosylase was purchased from New England Biolabs (Ipswitch, MA), piperidine and acrylamide were purchased from Fisher Scientific (Waltham, MA). Sodium cyanoborohydride and other chemicals were purchased from Sigma-Aldrich (St. Louis, MO).

### Cross-linking reactions

DNA duplexes were generated by mixing a ^32^P-labeled 2′-deoxyuracil-containing oligonucleotide with a slight excess of the complementary strand in MOPS (50 mM, pH 7) containing NaCl (100 mM), warming the mixture to 90 °C, followed by cooling to room temperature (24 °C). The AP site was generated by treatment with uracil DNA glycosylase (UDG, 10 units per mL final concentration) in a solution composed of Tris–HCl (20 mM, pH 8 @25 °C), dithiothreitol (1 mM), EDTA (1 mM), MOPS (25 mM, pH 7) and NaCl (10 mM) for 40 min at 37 °C. The DNA was ethanol precipitated and the duplex redissolved in a solution composed of sodium acetate (750 mM, pH 5.2) buffer containing NaCNBH_3_ (250 mM). After incubation for 24 h at 37 °C, the DNA was ethanol precipitated, redissolved in formamide loading buffer, and the products analyzed by electrophoresis on a denaturing 20% polyacrylamide gel. Following separation, the ^32^P-labeled oligonucleotides in the gel were visualized by phosphorimager analysis.

To verify that UDG treatment successfully generated AP sites in the strands, the oligonucleotides were heated at 90 °C in a aqueous piperidine (100 mM) for 30 min to induce strand cleavage at the AP site. The resulting DNA was dried in a Speed-Vac concentrator, redissolved in formamide loading buffer, and subjected to gel electrophoretic analysis. In the time-course experiments, aliquots of the reaction mixture were removed at selected time-points and frozen in dry ice prior to gel electrophoretic analysis. In the experiments involving mixtures of WT and mutant KRAS sequence, the ^32^P-labeled AP-containing probe strand was added to mixtures containing various ratios of WT and mutant KRAS sequences and processed as described above. Footprinting experiments to pinpoint the location of cross-link attachment on the target strands were conducted as described previously.^31,42^ The error bars shown in the figures reflect the standard deviation of the mean. Typical cross-link reactions were carried out in triplicate using a single batch of labeled probe. The cross-linking reactions in duplexes W and X were carried out in triplicate using at least four different batches of labeled probe (*i.e.* at least twelve separate cross-linking reactions). The slightly larger standard deviations observed for duplexes W and X are due to the fact that variation in cross-link yields between batches of labeled probe were somewhat greater than the variation in cross-link yields observed in triplicate repeats using a single batch of labeled probe. This may reflect batch-to-batch variations in salt impurities or specific activities associated with the labeled, AP-containing probe strands.

## Conflicts of interest

There are none to declare.

## Supplementary Material

RA-009-C9RA08009K-s001
