# Supplementary material for: Selective covalent capture of a DNA sequence corresponding to a cancer-driving C>G mutation in the KRAS gene by a chemically reactive probe: optimizing a cross-linking reaction with non-canonical duplex structures
Source: RSC Adv. 2019 Oct 15;9(56):32804–10. doi: 10.1039/c9ra08009k (PMC9073178; doi:10.1039/c9ra08009k)
Supplement: RA-009-C9RA08009K-s001 [file RA-009-C9RA08009K-s001.pdf]

Electronic Supplementary Material (ESI) for RSC Advances.
This journal is © The Royal Society of Chemistry 2019

Supporting Information for

Selective covalent capture of a DNA sequence corresponding to a cancer-driving C>G mutation in the KRAS gene by a chemically reactive probe: optimizing a cross-linking reaction with non-canonical duplex structures

Xu Guo,[‡] Maryam Imani Nejad,[‡] Li-Qun Gu[†,*] and Kent S. Gates[‡,§,*]

[‡] Department of Chemistry, University of Missouri, 125 Chemistry Building
Columbia, MO 65211

[§] Department of Biochemistry, University of Missouri, 125 Chemistry Building
Columbia, MO 65211

[†] Department of Bioengineering and Dalton Cardiovascular Research Center,
University of Missouri, Columbia, MO 65211

To whom correspondence should be addressed:
*Kent S. Gates: Tel.: (573) 882-6763; E-mail, gatesk@missouri.edu and
*Li-Qun Gu: Tel.: (573) 882-2057; E-mail, gul@missouri.edu

Table of Contents

Figure S1. Sequences used in this study..…....….S3

Table S1. Yields and selectivities of covalent capture of mutant and WT *KRAS* sequences by various AP-containing probes..S4

Figure S2. Bar graph comparing the yields and selectivities of covalent capture of mutant and WT *KRAS* sequences by various AP-containing probes...S5

Figure S3. Iron-EDTA footprinting reactions provide evidence that cross-link attachment in duplex W takes place at the guanine mutation in the nc35C>G *KRAS* gene sequence ……..S6

Figure S4. Time course for cross-link formation in duplex W.......................................S7

Figure S5. pH effects on the yield and selectivity of probe 12……...............................S8

Figure S6. Iron-EDTA footprinting the cross-link between probe 12 and the WT *KRAS* gene sequence in duplex X involves cross-link formation at the directly-opposing adenine residue……..S9

Figure S1

Mutant sequence nc35C>G: 5′-TGCCTACGCCAGCAGCTCCAA-3′
Wild-type sequence 5′-TGCCTACGCCACCAGCTCCAA-3′
Probe 1..5′-TTGGAGCTGCXGGCGTAGGCA-3′
Probe 2..5′-TTGGAGCTGAXGGCGTAGGCA-3′
Probe 3..5′-TTGGAGCTGCXTGCGTAGGCA-3′
Probe 4..5′-TTGGAGCTGCXCGCGTAGGCA-3′
Probe 5..5′-TTGGAGCTGCXAGCGTAGGCA-3′
Probe 6..5′-TTGGAGCTGCXGCGTAGGCA-3′
Probe 7..5′-TTGGAGCTGCXCGTAGGCA-3′
Probe 8..5′-TTGGAGCTGCXACGTAGGCA-3′
Probe 9..5′-TTGGAGCTGCXTGGCGTAGGCA-3′
Probe 10...5′-TTGGAGCTGCXCGGCGTAGGCA-3′
Probe 11...5′-TTGGAGCTGCXGGGCGTAGGCA-3′
Probe 12...5′-TTGGAGCTGCXAGGCGTAGGCA-3′

X=AP site

Figure S1. Sequences of oligonucleotides used in this study. The location of the cancer-driving nc35C>G mutation site is underlined in the mutant and wild-type sequences.

Probe	Probe-Mut Duplex	Yield	Probe-WT Duplex	Yield	Description
**1**	**A**<br>5'GCXGG<br>3'CGACC	7.2±0.9	**B**<br>5'GCXGG<br>3'CCACC	1.7±1.1	Fully base-paired
**2**	**C**<br>5'GAXGG<br>3'CGACC	4.2±0.6	**D**<br>5'GAXGG<br>3'CCACC	2.6±0.4	Mispair w/target guanine
**3**	**E**<br>5'GCXTG<br>3'CGACC	12.5±2.9	**F**<br>5'GCXTG<br>3'CCACC	3.1±1.0	Mispair on 3'-side of AP
**4**	**G**<br>5'GCXCG<br>3'CGACC	20.3±0.8	**H**<br>5'GCXCG<br>3'CCACC	2.5±0.6	Mispair on 3'-side of AP
**5**	**I**<br>5'GCXAG<br>3'CGACC	36.1±1.2	**J**<br>5'GCXAG<br>3'CCACC	5.1±0.4	Mispair on 3'-side of AP
**6**	**K**<br>5'GCXGC<br>3'CGA CG<br>C	27.4±1.0	**L**<br>5'GCXGC<br>3'CCA CG<br>C	2.4±0.3	Bulge in target strand
**7**	**M**<br>5'GCX CG<br>3'CGA GC<br>CC	24.0±0.2	**N**<br>5'GCX CG<br>3'CCA GC<br>CC	5.4±0.2	Bulge in target strand
**8**	**O**<br>5'GCX AC<br>3'CGA CG<br>C	25.1±2.3	**P**<br>5'GCX AC<br>3'CCA CG<br>C	5.5±0.4	Bulge in target strand
**9**	**Q**<br>T<br>5'GCX GG<br>3'CGA CC	2.4±0.2	**R**<br>T<br>5'GCX GG<br>3'CCA CC	3.0±0.3	Bulge in probe strand
**10**	**S**<br>C<br>5'GCX GG<br>3'CGA CC	17.0±0.8	**T**<br>C<br>5'GCX GG<br>3'CCA CC	7.5±0.4	Bulge in probe strand
**11**	**U**<br>G<br>5'GCX GG<br>3'CGA CC	12.1±1.3	**V**<br>G<br>5'GCX GG<br>3'CCA CC	8.0±0.4	Bulge in probe strand
**12**	**W**, 37 °C<br>A<br>5'GCX GG<br>3'CGA CC	51.6±6.4	**X**, 37 °C<br>A<br>5'GCX GG<br>3'CCA CC	10.2±3.3	Bulge in probe strand
**12**	**W**, 24 °C	35.3±1.6	**X**, 24 °C	5.3±0.7	Bulge in probe strand

Table S1. Yields of covalent capture (cross-link formation) for various probes with sequences corresponding to nc35C>G variant (Mut) and wild-type (WT) *KRAS* gene sequences.

Figure S2

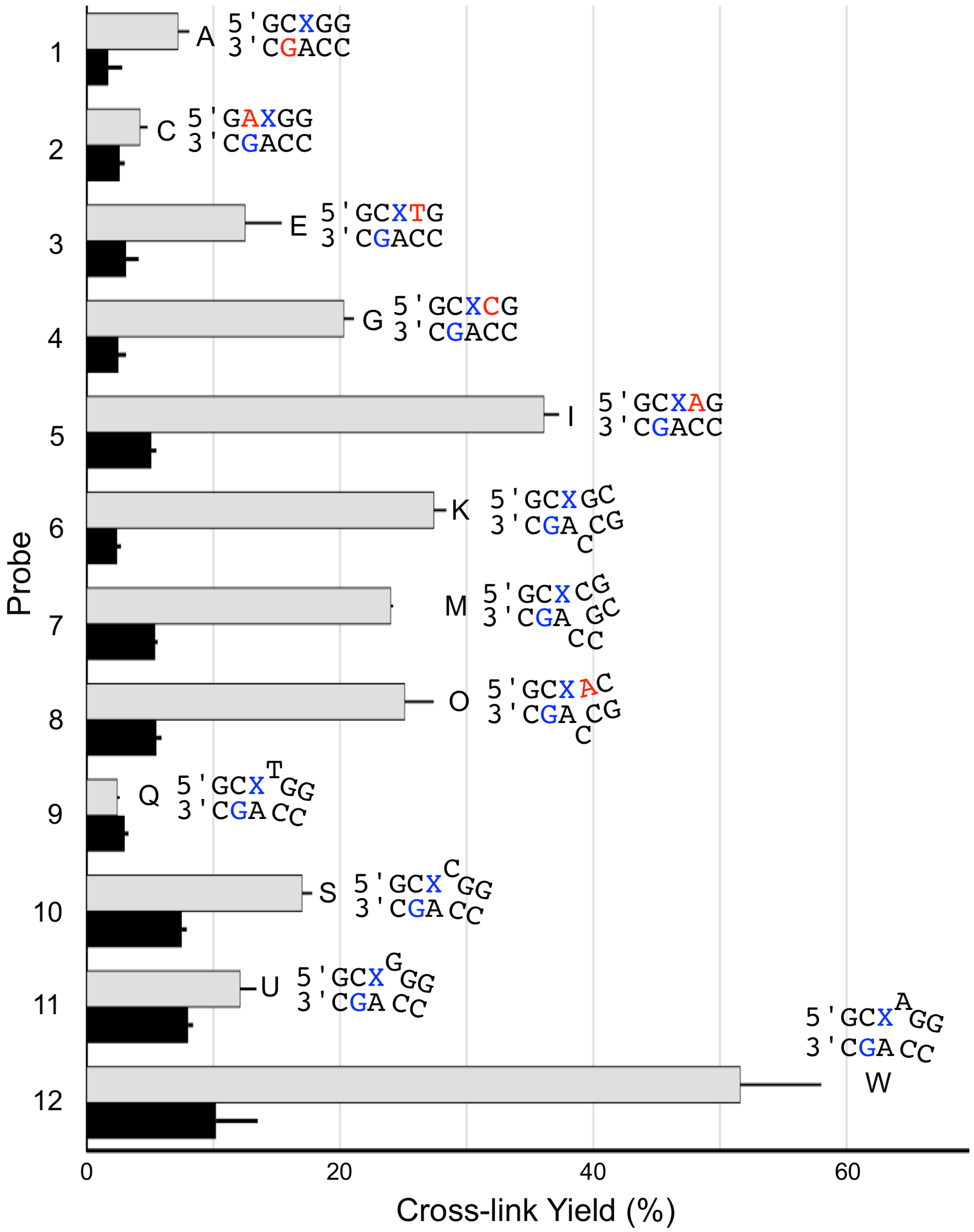

Figure S2. Bar graph comparing the yields and selectivities of covalent capture of mutant and WT KRAS sequences by various AP-containing probes. Probe-target complexes shown are the nc35C>G variant (Mut) of the *KRAS* gene sequence. Cross-link yields generated in the probe-mutant complexes are shown in the top bar of each pair (in gray) and the cross-link yields generated in the probe-WT (nc35C instead of G) KRAS sequence are shown in the lower bar of each pair (in black). The error bars depict the standard deviation calculated from at least three measurements.

Figure S3

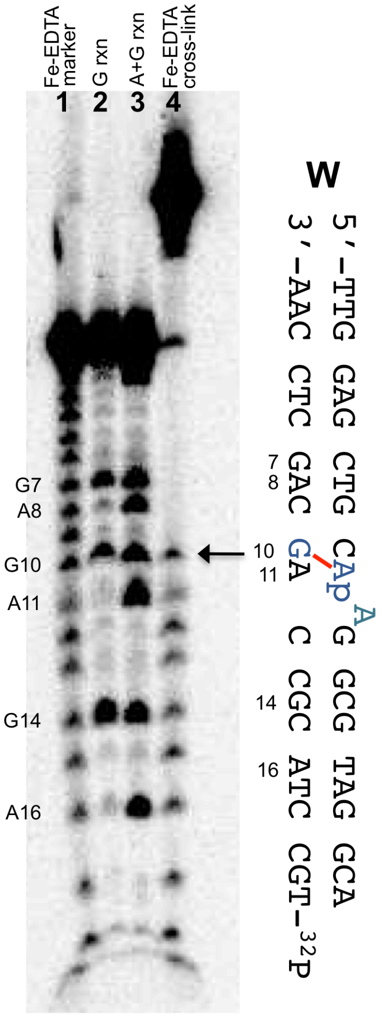

Figure S3. Iron-EDTA footprinting provides evidence that cross-link generation by probe 12 in duplex W involves attachment at the guanine mutation in the nc35C>G *KRAS* sequence. In this experiment, the site of cross-link attachment appears as the last band (marked with an arrow) before an interruption in the "ladder" of cleavage products generated by the iron-EDTA-H_2O_2 DNA-cleaving reagent, because cleavages beyond the cross-link yield large, slowly-migrating DNA fragments (seen in the upper part of lane 4) that are connected to the opposing strand (Luce, R. A.; Hopkins, P. B. *Methods Enzymol.* **2001**, *340*, 396-412). Lane 1: Fe-EDTA cleavage of the uncross-linked control; Lane 2: is a Maxam-Gilbert G-specific cleavage (sequencing) reaction on the 5'-^{32}P-labeled nc35C>G target strand; Lane 3 is an A+G specific cleavage (sequencing) reaction of the 5'-^{32}P-labeled nc35C>G target strand; Lane 4 is the hydroxyl radical footprinting reaction of the isolated probe-target duplex (^{32}P-labeled on the nc35C>G target strand). The ^{32}P-labeled oligodeoxynucleotides were resolved on a 20% denaturing polyacrylamide gel and visualized by phosphorimager analysis.

Figure S4

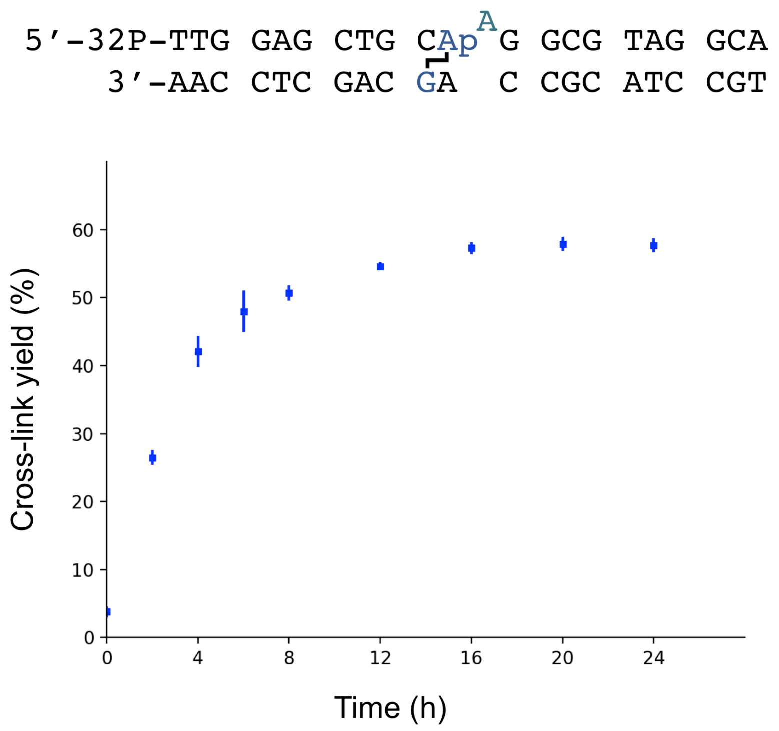

Figure S4. Time course for cross-link formation in duplex W. The probe-target duplex W was incubated in sodium acetate buffer (750 mM, pH 5) containing $NaCNBH_3$ (250 mM) at 37 ˚C. At various times aliquots were removed from the reaction, the DNA ethanol precipitated, and stored at –20 ˚C until electrophoretic analysis. The samples were dissolved in formamide loading buffer, loaded onto a denaturing 20% polyacrylamide gel and the DNA fragments resolved by electrophoresis. The ^{32}P-labeled oligodeoxynucleotides were resolved on a 20% denaturing polyacrylamide gel and visualized by phosphorimager analysis. The plot shows the percent yield of cross-link as a function of time.

Figure S5

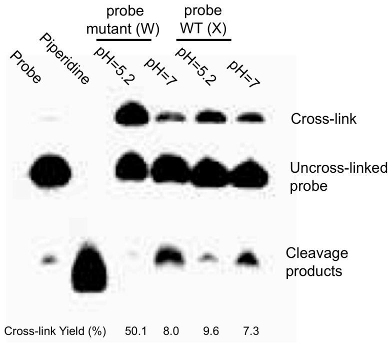

Figure S5. Effects of pH on the yield and selectivity of probe 12 for covalent capture of the mutant and wild-type *KRAS* gene sequences (duplexes W and X). Lane 1: labeled probe strand; lane 2: probe strand treated with piperidine to induce strand cleavage at the AP site (0.1 M piperidine, 30 min, 95 ˚C); lane 3: duplex W, sodium acetate (750 mM, pH 5.2) and $NaCNBH_3$ (200 mM); lane 4: duplex W, HEPES (50 mM, pH 7), NaCl (100 mM) and NaCNBH3 (200 mM); lane 5: duplex X, sodium acetate (750 mM, pH 5.2) and $NaCNBH_3$ (200 mM); lane 6: duplex X, HEPES (50 mM, pH 7), NaCl (100 mM) and $NaCNBH_3$ (200 mM).

Figure S6

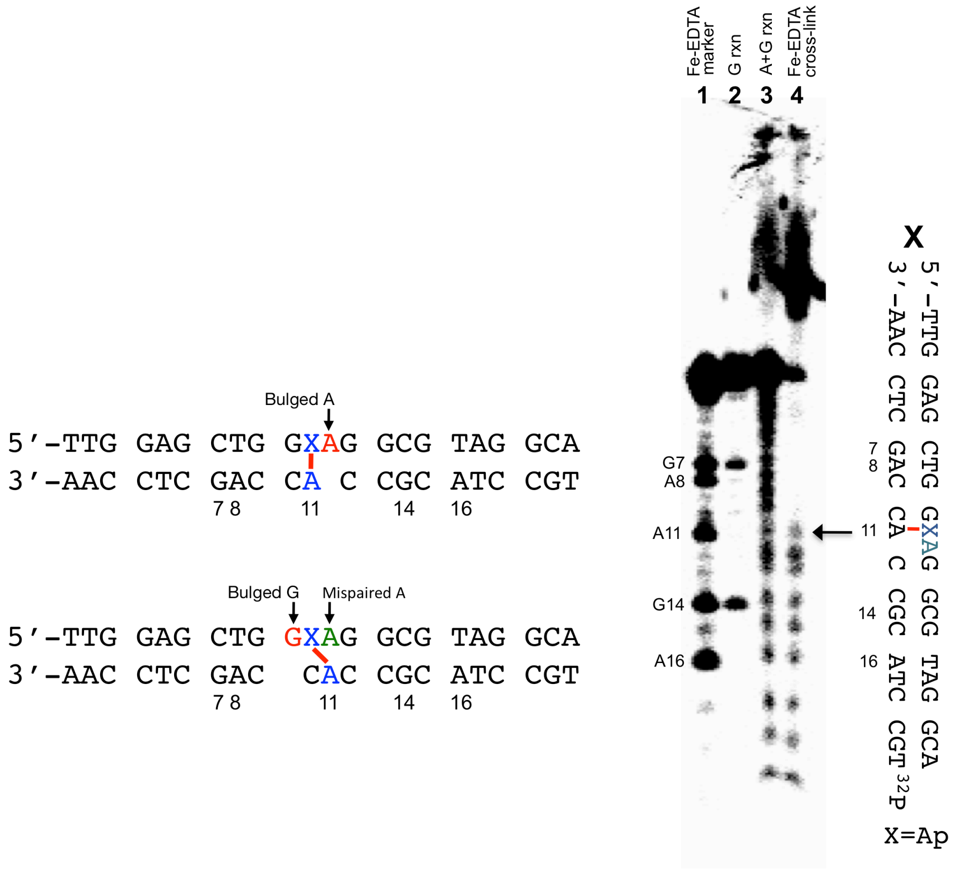

Figure S6. Iron-EDTA footprinting provides evidence that cross-link generation by probe 12 in duplex X involves attachment at the opposing adenine residue in the wild type (WT) *KRAS* sequence. In this experiment, the site of cross-link attachment appears as the last band (marked with an arrow) before an interruption in the "ladder" of cleavage products generated by the iron-EDTA-H_2O_2 DNA-cleaving reagent, because cleavages beyond the cross-link yield large, slowly-migrating DNA fragments (seen in the upper part of lane 4) that are connected to the opposing strand (Luce, R. A.; Hopkins, P. B. *Methods Enzymol*. **2001**, *340*, 396-412). Lane 1: Fe-EDTA cleavage of the uncross-linked control; Lane 2: is a Maxam-Gilbert G-specific cleavage (sequencing) reaction on the 5'-^{32}P-labeled WT target strand; Lane 3 is an A+G specific cleavage (sequencing) reaction of the 5'-^{32}P-labeled WT target strand; Lane 4 is the hydroxyl radical footprinting reaction of the isolated probe-WT duplex (^{32}P-labeled on the wild-type target strand). The ^{32}P-labeled oligodeoxynucleotides were resolved on a 20% denaturing polyacrylamide gel and visualized by phosphorimager analysis. The three-dimensional structure of the probe-WT duplex is not known. The probe-target complex has the potential to exist in (at least) two different forms (shown above, left). Cross-link formation in the WT KRAS sequence could proceed via a duplex with a bulged adenine residue (top left) or bulged guanine residue (bottom left). The complex with the bulged adenine places the target adenine residue into a potentially favorable arrangement for cross-link formation, similar to that seen in: Imani Nejad, M.; Shi, R.; Zhang, X., Gu, L.-Q.; Gates, K. S. *ChemBioChem*. **2017**, *18*, 1383-1386.
